# The complete chloroplast genome sequence of *Casuarina equisetifolia*

**DOI:** 10.1080/23802359.2021.1967803

**Published:** 2021-10-01

**Authors:** Jiaji Zhang, Yuhao Weng, Daiquan Ye, Yunfei You, Jisen Shi, Jinhui Chen

**Affiliations:** a Key Laboratory of Forest Genetics and Biotechnology of Ministry of Education of China, Co-Innovation Center for Sustainable Forestry in Southern China, Nanjing Forestry University, Nanjing, China; b National Germplasm Bank of Chinese fir at Fujian Yangkou Forest Farm, Nanping, China

**Keywords:** Chloroplast genome, *Casuarina equisetifolia*, Casuarinaceae

## Abstract

*Casuarina equisetifolia*, as windbreaks, soil erosion, and sand dune stabilization with high resistant to typhoon force winds, drought and salinization, belongs to the Casuarinaceae family. In this study, the complete chloroplast genome of *C. equisetifolia* was sequenced by Illumina sequencing platform and annotated by Geneious Prime. The complete chloroplast genome size is 156,128 bp in length, with a large single copy region (LSC: 86,192 bp) and a small single-copy region (SSC: 18,462 bp), which was separated by a pair of 25,737 bp inverted repeated regions (IRs). The chloroplast genome of *C. equisetifolia* encodes total 127 genes, including 82 protein-coding genes, 37 tRNA genes, and eight rRNA genes. The phylogenomic relationship analysis suggested that the Casuarinaceae family, which includes *C. equisetifolia*, was more closely related to the family of Betulaceae.

*Casuarina equisetifolia* is a member of the Casuarinaceae family and an invasive woody species, native to Malaysia, southern Asia, Australia, and Oceania (Hata et al. 2016). Nowadays, it has been successfully introduced to and gradually domesticated mainly in the southern Chinese coastal regions (Wei et al. 2021). It has high economic value due to providing fuel wood, land reclamation, dune stabilization, and scaffolding for construction, shelter belts, and pulp and paper production (Karthikeyan et al. 2013). However, there was very few genetic and genomic studies on the *C. equisetifolia*, which limits the study and utilization of *C. equisetifolia*. Herein, the complete chloroplast genome sequence of *C. equisetifolia* was assembled and characterized to provide more understanding of its evolution and genetic identification.

In this study, fresh leaves of *C. equisetifolia* were sampled from the Yangkou Forest Farm, in Fujian province, China, which located at 117.30–118.14 E, 26.39 − 27.12 N. The voucher specimen (voucher number MMH2020) was preserved at Key Laboratory of Forest Genetics and Biotechnology of Ministry of Education of China, Nanjing Forestry University. The total genomic DNA were extracted from fresh leaves and sequenced by a HiSeq Xten platform with the PE150 strategy (Novogene, Nanjing, China). After the sequencing, a total of ∼4.96 G raw data were obtained, then filtered and trimmed using SAMtools (Li 2011) and Fastp (Chen et al. 2018) to get the ∼4.94 G clean data. The chloroplast genomes *de novo* assembly was performed by software package velvet (Version. 1.2.10) (Zerbino et al. 2009) and annotated by Geneious Prime (Version. 2020.2.4) and tRNA-SCAN (Chan and Lowe 2019), with the chloroplast genome of putative closely related species *Alnus cremastogyne* (MH628453.1), *Betula alnoides* (MK888853.1) and *Carpinus putoensis* (KX695124.1) as references. The annotated complete chloroplast genome of *C. equisetifolia* was deposited in GenBank under the accession number of MZ032230.

A typical quadripartite structure was found in the complete chloroplast genome of *C. equisetifolia* with a length of 156,128 bp and ∼37% GC content. It contained a large single-copy region (LSC) of 86,192 bp with ∼34% GC content, a small single-copy region (SSC) of 18,462 bp with ∼30% GC content, and two inverted repeat regions (IRs) of 25,737 bp with ∼42% GC content. The genome annotation revealed that it contained total 127 functional genes, including 82 protein-coding genes, 37 tRNA genes, and eight rRNA genes.

Because of the lack of complete chloroplast genome data of species in the same family, 47 published species among the Fagales order, which *C. equisetifolia* resides within, were selected for a comprehensive phylogenetic analysis to determine the phylogenetic relationship of *C. equisetifolia*. Four species (*Licania canescens*, *Garcinia paucinervis*, *Suriana maritima*, and *Polygala japonica*) belonged to the Malpighiales and Fabales orders, which are the members of the Fabid clade together with the Fagales order, were used as outgroups. Fifty-one coding sequences (CDS) of protein-coding genes, which were commonly annotated in all 52 plants species, were selected and aligned using MEGA 7.0 (Kumar et al. 2016). The phylogenetic inference was generated based on maximum-likelihood (ML) analysis with the GTR model in RAxML v1.0.0 (https://raxml-ng.vital-it.ch/) (Kozlov et al. 2019). The phylogenetic tree showed that the members of Fagales order we selected has been divided into two groups. One group only contained the Fagaceae family, and the another one was consisted of the Betulaceae, Casuarinaceae, Fagaceae, Juglandaceae, Myricaceae family. In our study, the Casuarinaceae family, where only *C. equisetifolia* chloroplast was annotated in, was more closely related to the family of Betulaceae (Figure 1). The chloroplast genome sequence of *C. equisetifolia* in this study will be useful for further analysis on molecular markers and molecular breeding, and reminds us that the cognition of Casuarinaceae family is still very limited.

**Figure 1.**
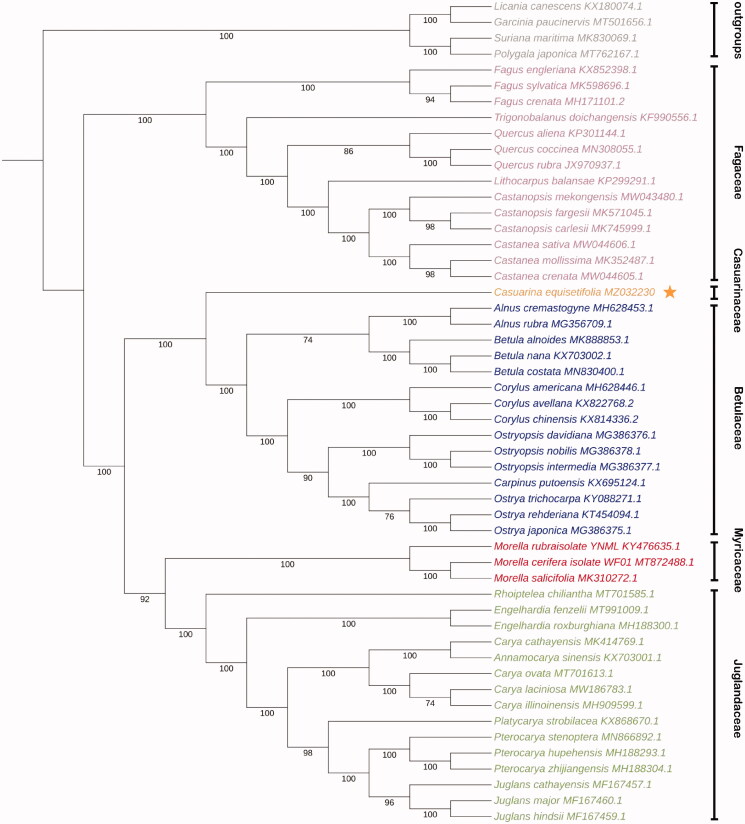
Maximum-likelihood (ML) tree based on CDSs of protein-coding gene of 47 plant species belonged to the Fagales order and four outgroups. Numbers below the branches represent the bootstrap support values (%). The Fagales order is subdivided in several plant families, indicated by color coding: the Betulaceae (blue), Casuarinaceae (orange), Fagaceae (purple), Juglandaceae (green), Myricaceae (red). Four outgroups are indicated in gray.

## Data Availability

The genome sequence data that support the findings of this study are openly available in GenBank of NCBI at (https://www.ncbi.nlm.nih.gov/) under the accession no. MZ032230. The associated BioProject, SRA, and Bio-Sample numbers are PRJNA726353, SRR14368234, and SAMN18928268, respectively.
